# Thermal Vibration-Induced Rotation of Nano-Wheel: A Molecular Dynamics Study

**DOI:** 10.3390/ijms19113513

**Published:** 2018-11-08

**Authors:** Haiyan Duan, Jiao Shi, Kun Cai, Qing-Hua Qin

**Affiliations:** 1 School of Forestry, Northwest A&F University, Yangling 712100, China; dhy@nwsuaf.edu.cn; 2 College of Water Resources and Architectural Engineering, Northwest A&F University, Yangling 712100, China; 3 Centre for Innovative Structures and Materials, School of Engineering, RMIT University, Melbourne 3001, Australia; kuncai99@163.com; 4 School of Engineering, the Australian National University, Canberra 2600, Australia

**Keywords:** carbon nanotube, nanomachine, thermal vibration, molecular dynamics

## Abstract

By bending a straight carbon nanotube and bonding both ends of the nanotube, a nanoring (or nano-wheel) is produced. The nanoring system can be driven to rotate by fixed outer nanotubes at room temperature. When placing some atoms at the edge of each outer tube (the stator here) with inwardly radial deviation (IRD), the IRD atoms will repulse the nanoring in their thermally vibration-induced collision and drive the nanoring to rotate when the repulsion due to IRD and the friction with stators induce a non-zero moment about the axis of rotational symmetry of the ring. As such, the nanoring can act as a wheel in a nanovehicle. When the repulsion is balanced with the intertubular friction, a stable rotational frequency (SRF) of the rotor is achieved. The results from the molecular dynamics simulation demonstrate that the nanowheel can work at extremely low temperature and its rotational speed can be adjusted by tuning temperature.

## 1. Introduction

A nanomachine is a device that works on the nanometer scale. By transforming the internal chemical or external physical energy, the nanomachine can generate power for specified functions in certain environments [1,2]. In 2016, three scientists won the Nobel Prize in Chemistry due to their significant contribution to the development of machines at molecular level [3]. The nanomotor is a key component of a nanomachine. Based on motion types, nanomotors can be classified into two groups: translation motors [4,5] and rotary motors [6,7,8,9,10,11]. For example, by placing a long inner tube in a short carbon nanotube (CNT) subjected to thermal gradient along the tube axis, Barreiro et al. [5] measured both translation and rotation of the shorter CNT with respect to the long tube. The rotary nanomotor developed by Fennimore et al. [10] and by Bourlon et al. [12] should also be mentioned. In their nanomotor, the metal plate attached on the CNT-shaft can be driven to rotate by external electric field. However, the size of these devices in at least one direction is far larger than that of a molecular machine in the traditional concept [13]. Hence, such “big” devices are usually not considered as “nanomotors”.

It is a challenge to physically fabricate a nanomotor with the size of a few nanometers. Fortunately, molecular dynamics simulation [14,15], which is based on the statistical physics and Newton’s laws of motion, is a powerful tool for the design and performance prediction of a nanodevice whose size is less than 10 nm. The numerical experiments have been widely applied in the design of nanostructures [16] or devices such as rotary nanomotors [17,18,19,20,21,22] and nano-strain sensors [23,24,25]. On the other hand, multi-walled carbon nanotubes (MWNTs) are usually adopted in the design of nanomotors due to the extremely low coefficient of intertubular friction and high strength of the nanotube. For example, by way of the molecular dynamics approach, Kang and Hwang [6] investigated the dynamic response of a CNT motor driven by nano liquid. In 2014, Cai et al. discovered a new rotary nanomotor made of CNT [9]. In their nanomotor, the rotor can be driven to rotate at 100GHz level by the fixed outer tube due to the thermal vibration of the atoms attached on the tubes. The rotation of the rotor in such rotary nanomotor can now be controlled accurately [17,19]. Recently, Cai et al. [20,21] proposed a method for measuring the rotation of the rotor of a nanomotor reported in [17,19].

It is noted from the review above that the CNT-based rotor is often actuated to rotate along its tube axis only. When we design a wheeled nanovehicle by mimicking a car, such tubular nanomotor cannot drive the nanovehicle to move. In general, a rotation transmission system [22], acting as a gear-box, is required to transform the rotation into linear motion of the vehicle. However, this will make the nanosystem more complex in terms of fabrication. To overcome the difficulty, a tyre-shaped nanoring by bending a CNT [26,27,28,29,30,31] into a wheel in constructed in the present work. The wheel is driven to rotate by fixed short CNTs at room temperature. When the short CNTs are assembled on a vehicle, the ring will work as a wheel. The dynamic behavior of the nanowheel is evaluated by molecular dynamics simulations and the related results are given in the following sections.

## 2. Numerical Tests and Discussion

### 2.1. Rotation of Rotor in the Stators without Relaxation

In this section, both the rotor and stators are formed from straight tubes via geometrical mapping method. The stators are fixed without relaxation. From the discussion in the section above, we know that the rotor will shrink slightly after being released in the NVT ensemble at T = 300 K. Hence, the interaction between the rotor and stators behaves as repulsion in the internal contact area, which will enhance the intertubular friction. In the following, we investigate the dynamic response of the three (S-, M-, and L-) types of rotors confined with different stators.

#### 2.1.1. S-Type Rotor

**Figure 1:**
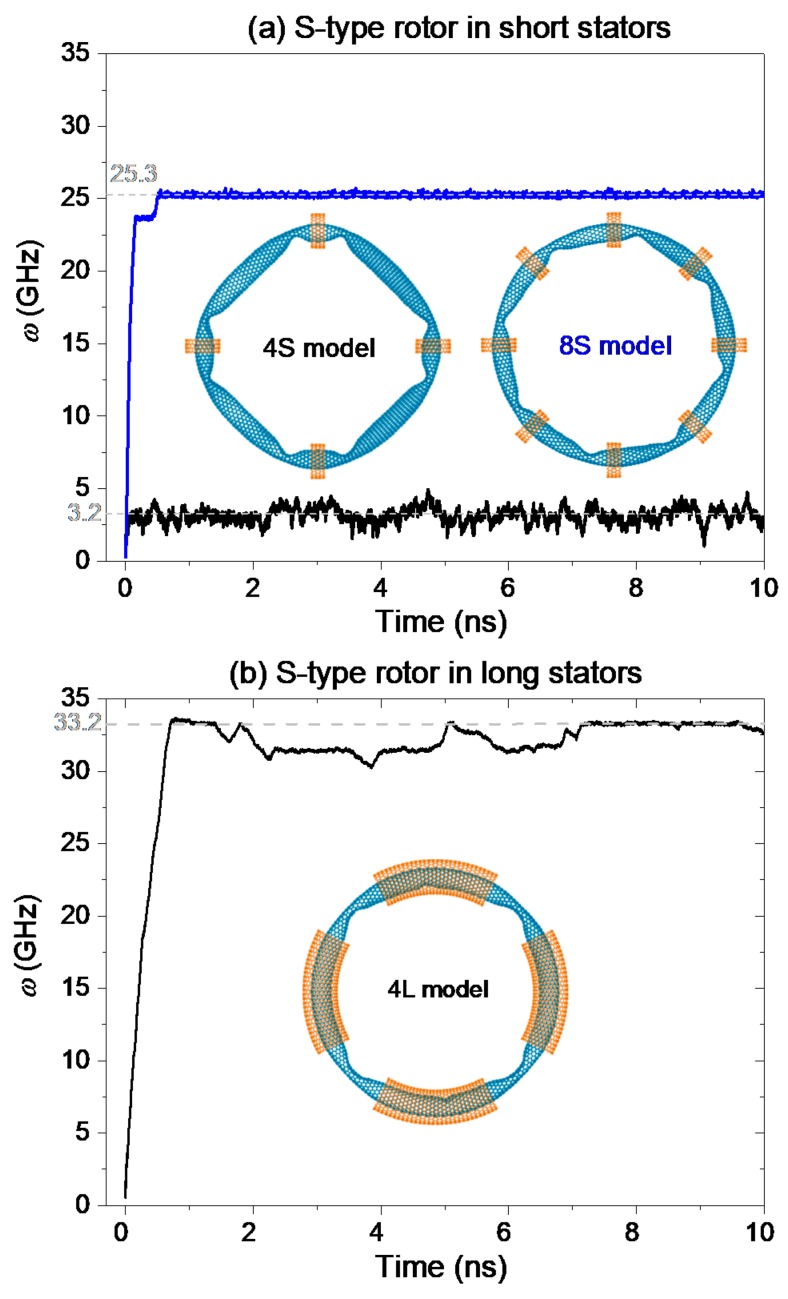
History curves of the rotational frequency (*w*) of the S-type rotor in different models at 300 K. (**a**) The rotor is confined with short stators. The two inset figures show the stable configurations of the nanowheels. Each rotor has eight depressions. (**b**) The rotor is confined with long stators, and the stable configuration of the system is inserted.

The curves displayed in Figure 1 demonstrate that the S-type rotor with radius of ~6.373 nm (Table 1) can be driven to rotate. However, the value of the rotor’s SRF depends on both the number and length of stators. For an S-type rotor in the 4S model (with four short stators), our calculation shows that its SRF can reach ~3.2 GHz within few picoseconds. We also found that a depression on the rotor varies slightly after a period of time’s running, and the depressions do not pass through the stators. Hence, the local buckling of the small rotor being in a status of local buckling can still be driven to rotate, but the continuous deformation of the depressions needs to overcome the surface potential barrier of the rotor [32,33]. This is also the reason for the slower rotation of the rotor in the model.

When we confine the S-type rotor with eight short stators, the SRF of the rotor is ~25.3 GHz, which is greater than that of the rotor in the 4S model. Two major reasons lead to this result. One is that the number of stators is twice of that in the 4S model. According to the definition of *w* in Equation (2), the rotational frequency is proportional to the number of stators *N*_s_. However, even to be twice of 3.2 GHz, the value of SRF is still far less than 25.3 GHz. So, we conclude that the friction between the rotor and stators is reduced when the number of the short stators increases. The other is that the friction between rotor and stator is reduced because the rotor has small curvature at a depression when more stators are used to confine the rotor. It can be verified by comparing the two inset pictures in Figure 1a.

By putting the rotor in four long stators, the SRF of the rotor can be larger than 25.3 GHz. Figure 1b indicates that the SRF of the S-type rotor in the 4L model reaches 33.2 GHz. It is because the rotor has smaller deformation at eight depressions in the 4L model than in the 4S/8S model. The friction between the edges of stators and the depressed part on rotor becomes negligible. Meanwhile, the friction between the saturated carbon atoms in CNTs is extremely low [34,35,36]. Hence, the SRF of the rotor in the current model can be as large as 33.2 GHz.

#### 2.1.2. M-Type Rotor

**Figure 2:**
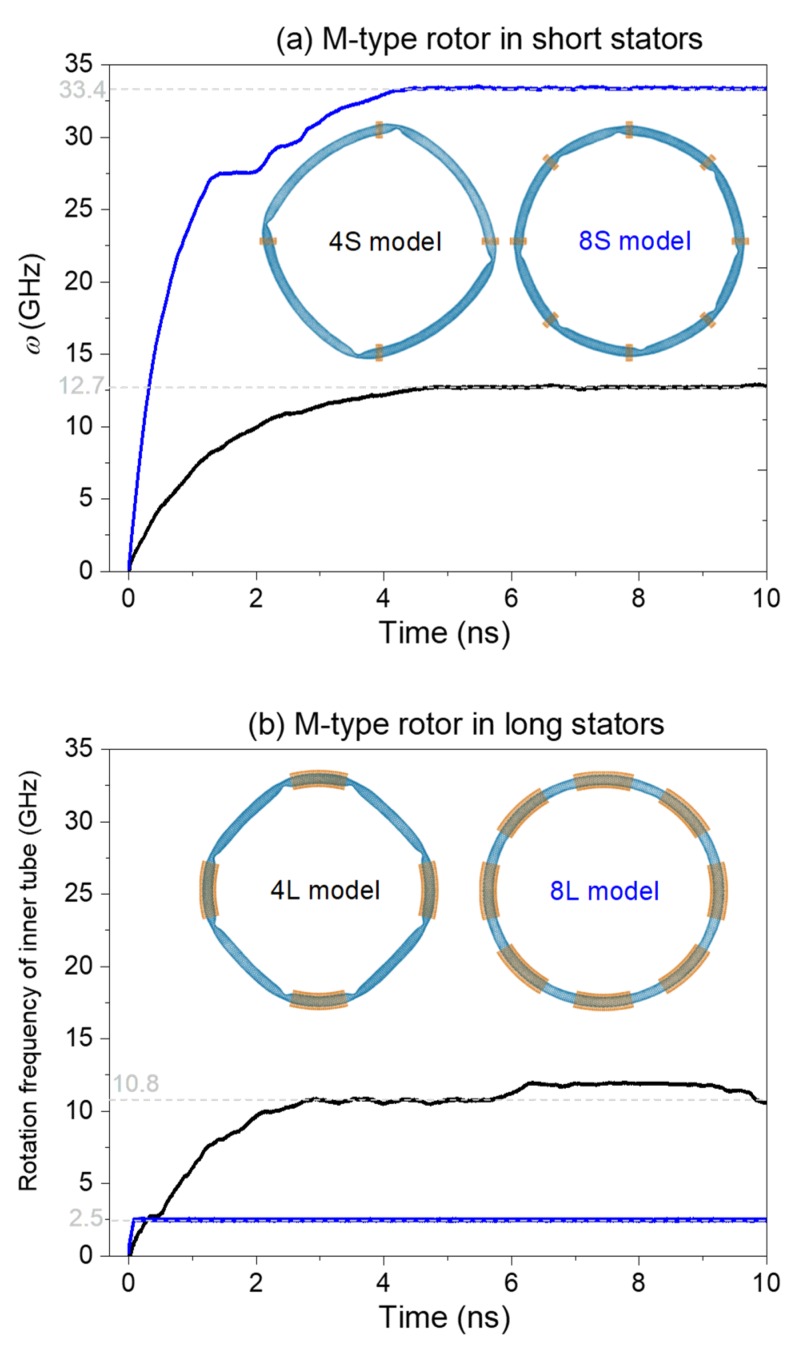
History curves of the rotational frequency (*w*) of the M-type rotor in different models at 300 K. (**a**) The rotor is confined with short stators. (**b**) The rotor is confined with long stators.

Consider a M-type rotor as shown in Figure 2. Its radius is twice as that of the S-type rotor (as can be seen in Table 1). Therefore, the buckling-induced deformation of the rotor becomes weaker than that of the S-type rotor. This will benefit the acceleration of the rotor. Figure 2 gives the history curves of the rotational frequency of the M-type rotor in different models. For example, in Figure 2a, the values of SRF of the rotor reaches ~12.7 GHz and ~33.4 GHz in the 4S model and 8S model, respectively. From the inserted configurations of the rotor in Figure 2a, the rotor has four depressions in the 4S model, but the deformation of the rotor is larger than that of the rotor in the 8S model. Hence, the SRF of the rotor in the 4S model is smaller.

However, when we use longer stators to confine the rotor, the value of SRF of rotor in either 4L model or 8L model in Figure 2b is smaller than 12.7 GHz. The rotor has large deformation near the edges of stators. Hence, the intertubular friction is larger and the continuous deformation of the depressions costs more energy, which results in a slower rotation of the rotor with SRF of ~2.5 GHz. When more stators are used, the value of SRF is increased to be as high as 10.8 GHz.

#### 2.1.3. L-Type Rotor

**Figure 3:**
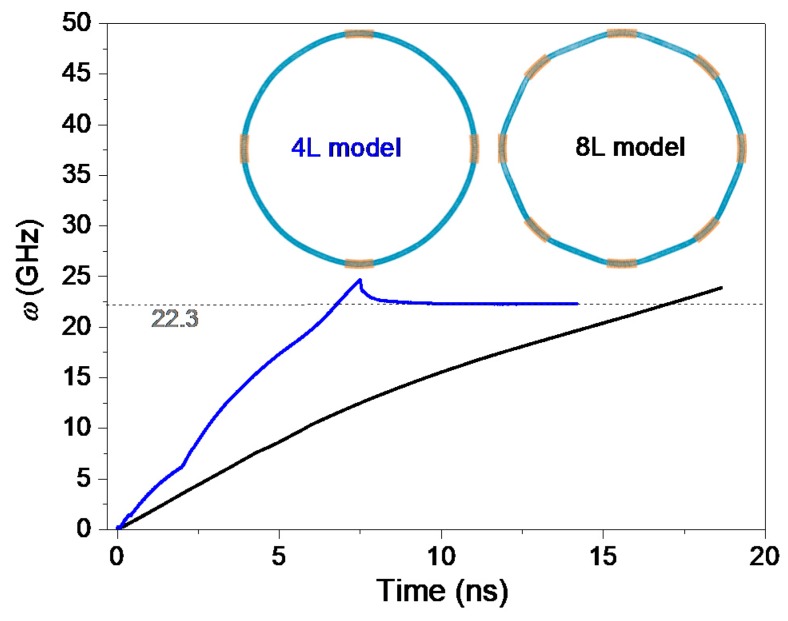
History curves of the rotational frequency (*w*) of the L-type rotor confined by long stators at 300 K.

To confine the L-type rotor with radius of ~25.493 nm, only long stators are used. Comparing to the S- and M-type rotors, the L-type rotor has larger compliance when confined with the same number of the same stators. Especially, when the short stators are used, the slim doughnut-shaped rotor has larger vertical deflection from its original position. Figure 3 shows that there is no local depression in the stable configuration of the rotor.

For the rotor in the 4L-type model, its SRF reaches 22.3 GHz after about 8 ns of acceleration. The rotor in the 8L-model needs longer time, saying over 18 ns, in acceleration because the rotational frequency keeps increasing after 18 ns. From Equation (2), the value of SRF depends on the number of stators. In the 8L-model, the number of stators is twice of the stators in the 4L-type model. It also means that the rotor in the 8L-type model needs shorter time on acceleration. The slopes of the two curves in Figure 3 are obviously contrary to the rule. Therefore, we conclude that the difference between ***F****_τ_* and ***F***_c_ is smaller in the 8L-type model due to more severe deformation of the rotor in the model.

### 2.2. Rotation of Rotor in the Stators After Relaxation

**Figure 4:**
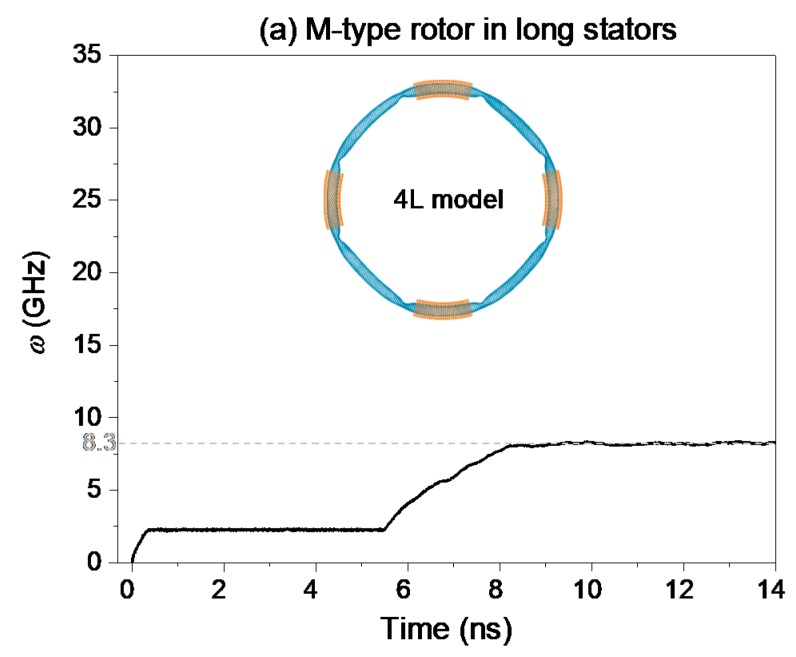

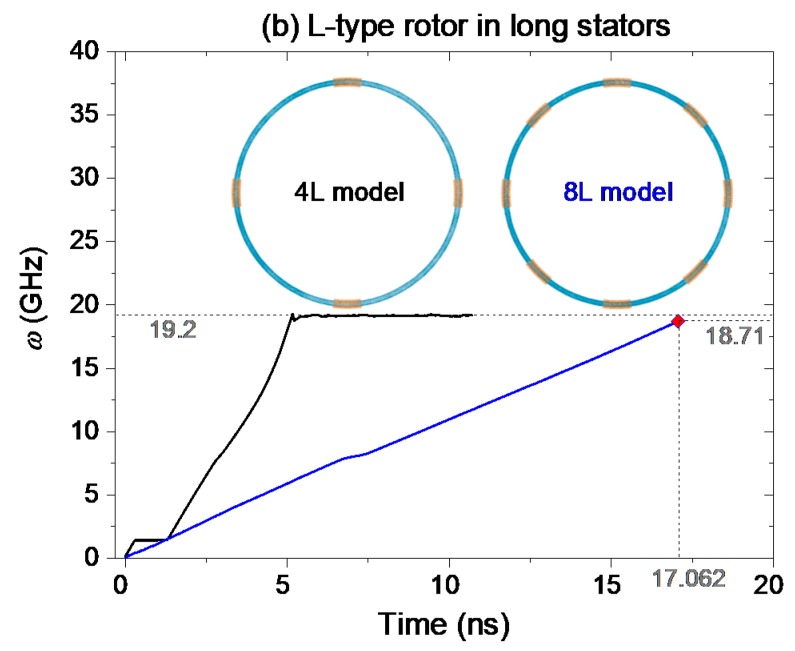
History curves of the rotational frequency (*w*) of the M-/L-type rotors confined by long stators after relaxation at 300 K. (**a**) M-type rotor. (**b**) L-type rotor.

In the analysis above, the rotors are confined by the stators without relaxation. As the rotor has a small shrink after relaxation, the rotor is under tension along its circular direction, and is subjected to repulsion from the stators. In this section, we reduce the pre-tension state of the rotor by confining the rotor after 100 ps of relaxation. The geometry used in the model is the same as that given in the previous sections. The temperature is assumed to be 300 K. Each stator has four atoms with the same IRD. The typical results are shown in Figure 4. From the inset figures in Figure 4, the rotors have smaller deformation than those in Figure 2b and Figure 3. Hence, the regulation of the stators is effective in controlling the configuration of the rotor.

In Figure 4a, the SRF of the rotor is ~8.3 GHz after ~8 ns of acceleration. Obviously, the value of SRF is smaller than that shown in Figure 2b. Hence, the interaction between the tubes influences the value of SRF. From Equation (2), if the friction between the rotor and the stators is larger due to reasons such as faster relative sliding and stronger thermal vibration of the rotor, the SRF of the rotor becomes smaller. If the collision between the rotor and the IRD atoms on the rotor is stronger, the value of SRF could be larger on the condition that the friction (***F***_c_) grows slower than the repulsion (***F****_τ_*). Obviously, smaller value of SRF in Figure 4a is mainly caused by weaker collision between the tubes.

Figure 4b verifies the prediction that the value of SRF becomes smaller due to weaker collision between the rotor and the stator after relaxation. Meanwhile, the acceleration of the L-type rotor driven by more stators becomes slower. The major reason is that the amplitude of vibration of the rotor with respect to temperature is reduced by more stators, resulting in weaker collision between the rotor and the IRD atoms.

### 2.3. Temperature Effect on Rotation of Rotor

**Figure 5:**
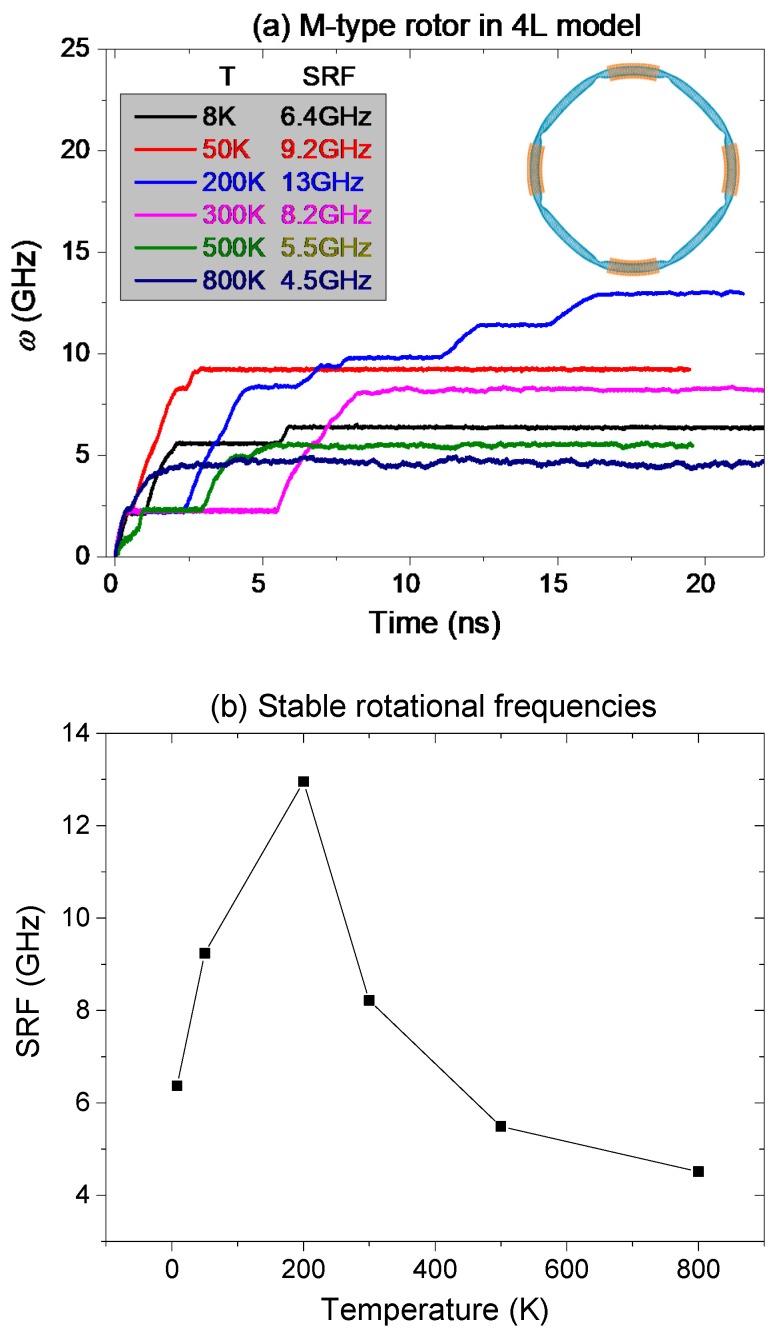
Temperature effect on rotational frequency of rotor. (**a**) History curves of the rotational frequency (*w*) of the M-type rotor confined by four long stators after relaxation at different temperatures. (**b**) Curve of stable rotational frequency (SRF) vs. Temperature for the M-type rotor.

As the rotation of the rotor is excited by the thermal vibration of atoms, the temperature of the system is the key factor determining the response of the rotor. To show the temperature effect on the SRF of the rotor, six values of temperature ranging from 8 to 800 K are employed in the discussion. The M-type rotor in the 4L model is adopted in numerical simulation. Each stator has four IRD atoms. The history curves of the rotational frequency of the rotor are given in Figure 5.

After a period of time’s acceleration, the SRF of the rotor becomes stable at different levels for different values of temperature. For example, at extremely low temperature, say 8 K, the SRF of the rotor is ~6.4 GHz. At 800 K, the rotor has a stable rotation with frequency of ~4.5 GHz. Among the six cases, the value of SRF of the rotor at 200 K reaches its maximum. From Equation (2), the value of SRF depends on the difference between ***F****_τ_* and ***F***_c_. At lower temperature, the difference between the repulsion and friction is smaller due to weaker thermal vibration of the rotor. Hence, the SRF is smaller. At higher temperature, both the repulsion and friction between the tubes are larger, whilst the difference between them is smaller. Hence, at very high temperature, say 800 K, the SRF of the rotor is smaller, either. Only at the temperature near 200 K, the difference between the repulsion and friction is larger, resulting in larger value of SRF.

Two phenomena shown in Figure 5 are worthy of demonstration. One is that the rotor can be excited to rotate at extremely low temperature, which means that a nanovehicle with such nanowheel can work at such low temperature. The other is that the SRF of the wheel can be regulated by adjusting the temperature. It hints a wider potential application of the nanodevice.

### 2.4. Effect of IRD Schemes on Rotation of Rotor

#### 2.4.1. Different Layout of IRD Atom(s)

**Figure 6:**
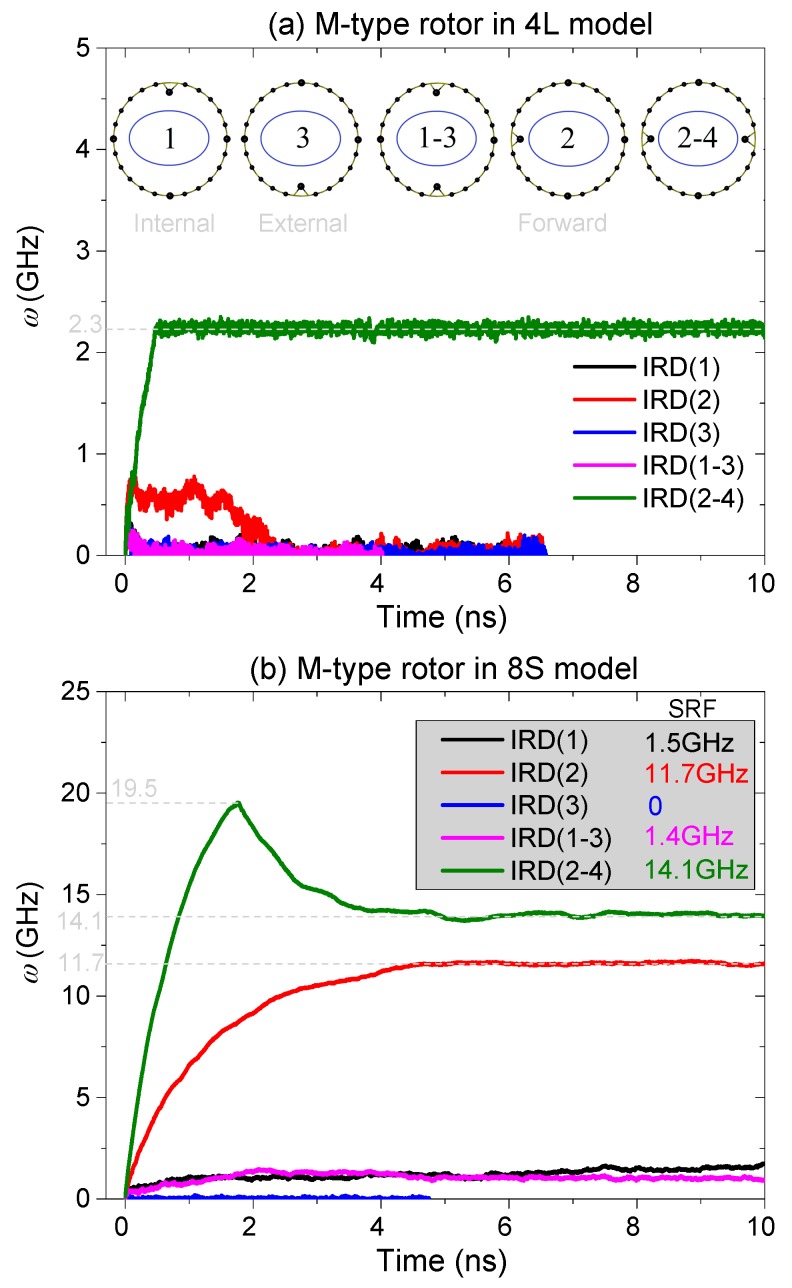
History curves of the rotational frequency of the M-type rotor driven by different stators at 300 K (**a**) In 4L model, (**b**) in 8S model.

**Figure 7:**
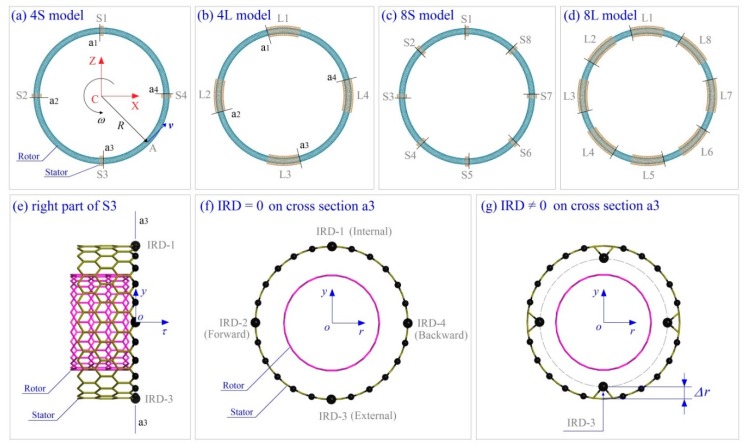
Geometry of rotary nanowheels from bent carbon nanotubes (CNTs). (**a**) The 4S model for a nanowheel having a rotor (i.e., tyre-shaped ring) and four short stators labeled as S1, S2, S3, and S4, respectively. (**b**) The 4L model having a rotor and four long stators labeled as L1, L2, L3, and L4, respectively. (**c**) The 8S model. (**d**) The 8L model. *R* is the average radius of ring. ***v*** is the velocity of the atom A on the ring, and *w* is the average rotational frequency of the ring. XYZ is the global coordinate system with origin at the center of ring (point C). The local coordinate system is *τry* with origin at the center of inner tube′s cross section. (**e**) Right part of the third short stator (S3) in (**a**). The black balls are the unsaturated edge atoms on S3. (**f**,**g**) Only at the right-end cross section (a3) of S3, the four large black atoms with inwardly radial deviations are labeled as IRD-1, IRD-2, IRD-3, and IRD-4, respectively. Other stators do the same. The four inwardly radial deviation (IRD) atoms have the same IRD, e.g., *∆r* = 0.4 × *l*_C-C_ = ~0.0568 nm.

From the mechanism for the thermally driven rotation of the nanowheel discussed above, the rotation of the doughnut-shaped rotor is driven to rotate due to the collision with the IRD atoms on stators. In general, more drastic collision leads to stronger repulsion which generates the rotational acceleration of the rotor along Y-direction, and further results in larger value of SRF of the rotor are given in Equation (2). It should, however, be mentioned that the collision between the rotor and the IRD atom also depends on the location of the IRD atom because the thermal vibration of the atoms on the rotor is not symmetric along the *τ*-axis because of the curvature of the rotor toward internal side of the doughnut. To illustrate the contributions of each IRD atom on driving the rotation of the rotor, we present five layout schemes of IRD atoms in every stator. The M-type rotor is confined with four long stators. In each scheme, a stator has one or two IRD atoms, and the layout schemes are inserted in Figure 6a. For example, when only the internal IRD atom (Figure 7a) is used, the scheme is labeled as “IRD(1)”. If only the external IRD atom is used, the scheme is labeled as “IRD(3)”. In general, the distance between the rotor and the internal IRD atom (IRD(1)) is smaller than that between the rotor and the external IRD atom. If both the internal and the external IRD atoms are used, the scheme is labeled as “IRD(1–3)”. As the forward and backward IRD atoms are in symmetrical layout, only one of them e.g., the IRD(2) scheme, is discussed. The “IRD(2–4)” scheme is also discussed when both the forward and the backward IRD atoms are used. The history curves of the rotational frequency of the M-type rotor in different models are shown in Figure 6.

Figure 6a shows that the SRF of the rotor tends to be zero except in the scheme of IRD(2–4), i.e., the rotor is driven by both of the 2nd (forward) and the 4th (backward) IRD atoms on an edge of a stator. The SRF of the rotor is ~2.3 GHz in the scheme of IRD(2–4). From the pink curve shown in Figure 5, we know that the SRF of the rotor is ~8.2 GHz when each stator has four IRD atoms. So, the present scheme of IRD atoms (IRD(2–4)) in each stator only provides weaker repulsion on the rotor and results in smaller value of SRF. If there is only one IRD atom on each stator, the rotor cannot be excited to rotate. Two reasons lead to the phenomenon. One is that the cross section of the M-type rotor looks like an ellipse, whose long axis is aligned with the normal of XZ-plane. Hence, the 1st and 3rd IRD atoms are far away from the rotor’s surface as comparing to the second and the fourth IRD atoms. During the thermal vibration of the rotor, the collision between the rotor and the 1st or the 3rd IRD atoms, or both, becomes too weak to actuate the rotation of the rotor. Another reason is that the collision between the rotor and the 1st and the 3rd IRD atoms mainly depends on the breath-like vibration of the rotor within XZ-plane. The frequency of the break-like vibration is much lower than that of the atom’s thermal vibration. Hence, the repulsion from the two IRD atoms is weak. However, when both the 2nd and the 4th IRD atoms exist, the 1st and the 3rd IRD will improve the collision between the rotor and the IRD atoms, and further produce stronger repulsion. A conclusion can be made that the position of an IRD atom can influence the value of the rotor’s SRF of the rotor. Briefly, the forward and the backward IRD atoms can provide stronger repulsion on the rotor than the internal and external IRD atoms, and result in larger SRF.

When we use 8 short stators to confine the rotor, and each stator has one or two IRD atoms as shown in the inserts in Figure 6a, the rotor can be driven to rotate easier than that confined with 4 long stators. From Figure 6b, the SRF of the rotor is zero only when the 3rd IRD atom exists in each stator. The reason is that the 3rd IRD atom is at the external edge of a stator, and provides no repulsion to the rotor. However, the 1st IRD atom is at the internal edge of a stator, it provides strong repulsion on the rotor which has large deformation when confined with short stators (the 8S model in Figure 2a). Therefore, when both the 1st and the 3rd IRD atoms exist, the SRF of the rotor is approximately equal to that of the rotor when driven only by the 1st IRD atom. If the 2nd or the 4th IRD atoms, or both, exist, the SRF of the rotor is over 10 GHz.

#### 2.4.2. Different Number of IRD Atoms

**Figure 8:**
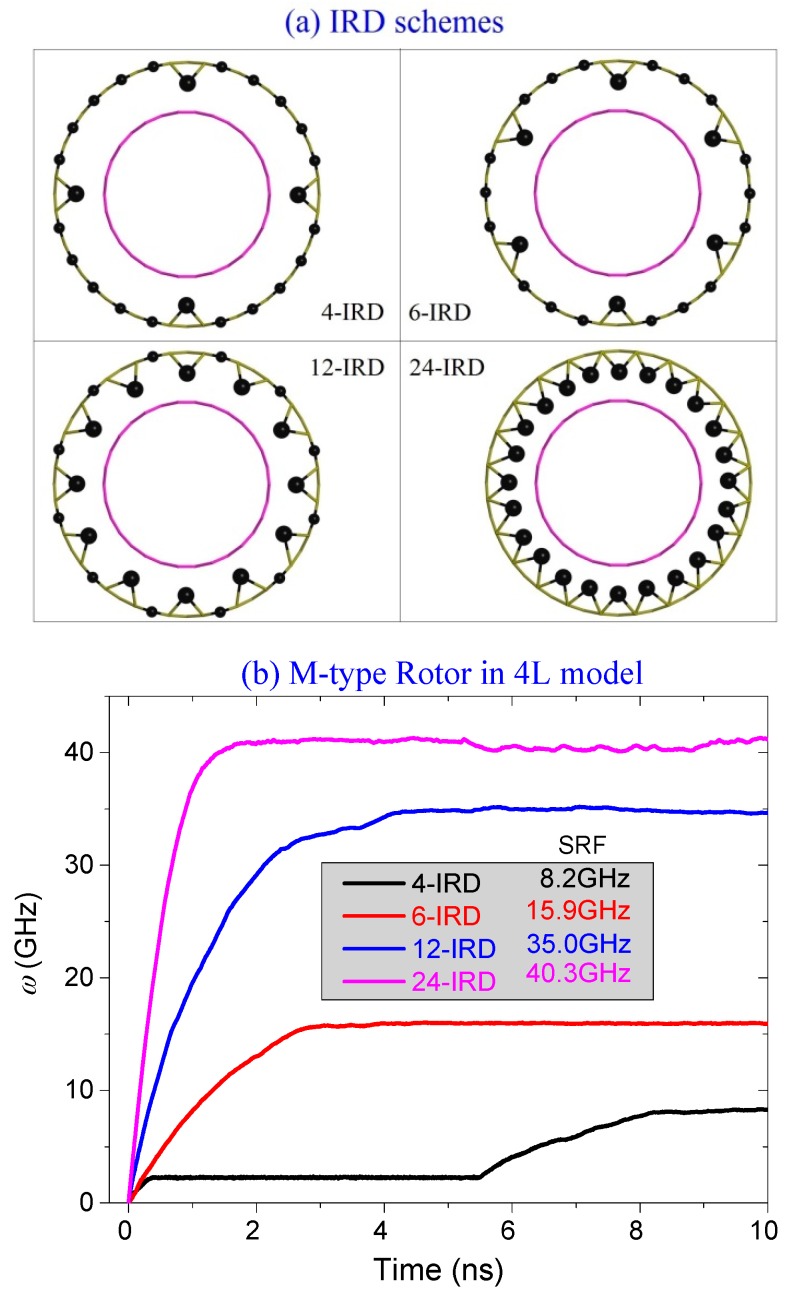
The histories of the rotational frequencies of the rotor driven by different number of IRD atoms on each stator. (**a**) Four IRD schemes. (**b**) Rotational frequencies with respect to four IRD schemes.

Can we obtain a rotor with higher value of SRF if more atoms are set on one edge of each stator with IRD? Figure 8 presents the answer. Besides the original 4-IRD scheme, i.e., each stator has four IRD atoms, we choose three other schemes to show the effect of number of IRD atoms on the value of SRF. The details are shown in Figure 8a. Figure 8b illustrates the values of the SRF of the rotor in different cases. For example, when there are six IRD atoms on each stator, the value of SRF is ~15.9 GHz, which is almost twice of that with respect to the 4-IRD scheme. When half of the edge atoms are set to have IRD, i.e., 12-IRD scheme, the value of SRF reaches 35 GHz, which is more than four times of that with respect to the 4-IRD atom. But the value of SRF increases only ~5.3 GHz from 35 GHz when all the edge atoms have IRD, i.e., 24-IRD scheme. One conclusion can be drawn that more IRD atoms distributing uniformly on an edge of each stator result in the larger value of SRF of the rotor. Clearly, the SRF of the rotor has a maximum value when all edge atoms have IRD.

## 3. Models and Methodology

### 3.1. Models of Nanowheels

Consider a four nanowheel model as shown in Figure 7a–d. The rotors are made from CNT (15, 0) with different lengths, and the stators from CNT (24, 0) with different lengths as well. The radii of the two tubes are ~0.5872 and ~0.9395 nm, respectively. The other parameters of the model are listed in Table 1. The IRD atoms in Figure 7g are in the cross section in the local *y-r* plane. In each stator, only one edge has IRD atoms, i.e., on cross section a1, a2, a3, or a4 as shown in Figure 7a,b.

### 3.2. Reference Frames

To show the relative positions of key atoms in the system, two types of reference frames are used. The fixed Cartesian coordinate system C-XYZ is the global reference frame, whose origin is at the center of the system, point C. Y-axis (not labeled) is vertical to the plane, and is the axis of the rotational symmetry of the initial ring. The neutral layer of the ring locates in plane XZ. A local frame labeled *o-τry* is used to illustrate the relative position of an atom in the longitudinal section of the rotor. The local origin, point *o*, is on the curved tube axis of the ring. The local axis “*or*” points to the global origin. The local axis “*oy*” is vertical to the global plane XZ. The local axis “*oτ*” is the tangent direction of the curved tube axis of the rotor.

### 3.3. Flowchart of Molecular Dynamics Simulation

Molecular dynamics simulations are used to investigate dynamic behaviour of the nanowheel shown in Figure 7a–d. The simulation is performed using the open source code LAMMPS [37]. In each simulation, the update of the positions of atoms are determined by time integration of the Newton’s second law of motion, in which the resultant force of each atom is obtained by means of the partial derivatives of the potential energy of the atom with respect to its coordinates. The potential energy of each atom is calculated by substituting the relative positions of its neighbor atoms in the AIREBO potential [38]. The cutoff of the non-bonding interaction, e.g., van der Waals interaction [39], among atoms is set to be 1.02 nm, and the timestep is 0.001 ps. The system is put in a canonical (NVT) ensemble with a constant temperature, and the velocities of the atoms are modified to match the given temperature every 100 steps. Nosé–Hoover thermostat [40,41] is used for the modification. In each simulation, following steps are carried out:

(1) Build a double-walled CNT with chirality of (15, 0)/(24, 0) and specified length;

(2) Cut the outer tube into four or parts with the same specified length, layout them uniformly on the inner tube;

(3) If necessary, relax the tubes at a NVT ensemble for 100 ps;

(4) Form the inner tube into a nanowheel by geometrical mapping method, and similarly deform the outer tube and set IRD atoms;

(5) Fix the atoms on the stators after minimization of potential energy of the system;

(6) Run at most 25,000,000 steps for updating the system in a canonical ensemble, and record data for further analysis;

(7) Stop.

### 3.4. Possible Configuration of Rotors after Relaxation

**Figure 9:**
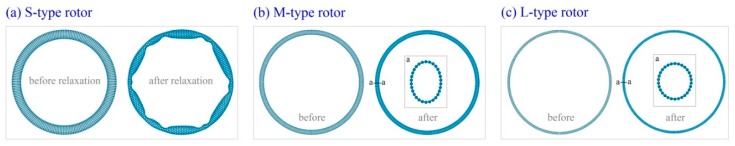
Configurations of a rotor before and after relaxation at 8 K. (**a**) The S-type rotor with radius of ~6.373 nm before relaxation or ~6.242 nm after relaxation. (**b**) The M-type rotor with radius of ~12.746 nm (before) or ~12.410 nm (after). The cross section at the a-plane is an ellipse. (**c**) The L rotor with radius of ~25.493 nm (before) or ~25.170 nm (after). The cross section at a-plane is approximately circle.

Figure 7a–d show the initial idea of the nanowheels constructed via geometrical mapping from one-dimensional tube to two-dimensional ring. After relaxation, the rotor may have further deformation due to large curvature of tube surface. After relaxation, the C-C bonds along tangent direction of the ring have obvious variation. For example, when the distance between the atoms and the origin of the global coordinate system is less than *R*, the bonds related to the atoms become shorter. It means that the bonds are under compression. Otherwise, the bonds are under tension. For a thin shell under compression, local buckling [42,43,44] may happen. For example, for the rotor after relaxation in Figure 9a, its internal surface has 12 depressions due to serious compression exceeding the critical buckling stress. When the radius of rotor becomes larger, e.g., Figure 9b, the rotor has no depression after relaxation. However, the ellipse-shaped cross section (labeled “a”) indicates that the tube is still under high compression, and local buckling may happen when an external load is applied upon it. For the cross section (a-plane) of the rotor after relaxation in Figure 9c, it deforms slightly, which means that the rotor is not easily under locally buckling state.

### 3.5. Propulsion of a Nanowheel within the NVT Ensemble

**Figure 10:**
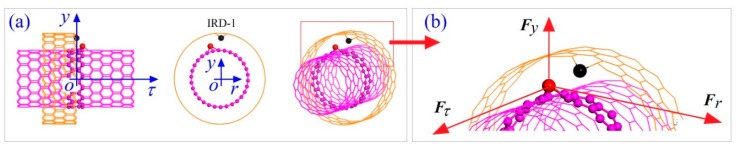
Schematic of transient interaction between the rotor and a short stator. (**a**) Three views of the local configuration of wheel. The *y*-axis is vertical to XZ-plane (Figure 7a). The red atom on rotor having a deviation from its equilibrium position indicates the thermal vibration of the atom. The black atom on stator with obvious deviation is the IRD atom. (**b**) The interaction between the IRD (black) atom and the red atom (within the red frame in (**a**)). The three components in the local coordinate system of the force exerted upon the red atom are labeled as ***F****_y_*, ***F****_r_* and ***F****_τ_*, respectively.

Figure 10 shows the schematic of the interaction between the rotor and the IRD atom on a short stator. For example, when the red atom on the rotor wall comes closer to the IRD (black) atom due to thermal vibration, there must have a repulsion exerted upon the red atom due to their distance being less than the balance distance (~0.34 nm). The repulsion has three components along the three axes of the local Cartesian coordinate system. The component ***F****_y_* will lead to the red atom move away from the IRD atom along the negative direction of y-axis. As the rotor is confined by the short stator, the part of the rotor located within the outer tube has to be bouncing frequently along y-direction. Similarly, for the component ***F_r_*** it will make the rotor bounce along the ***r***-direction. Obviously, when the two components, ***F****_y_* and ***F_r_***, can have a torque about the origin “*o*”, the rotor will rotate along *τ*-axis. It can be expressed mathematically as

(1) $$\omega_{\tau }=N_{s}\times \int_{0}^{t}\frac{r\times \left(F_{y}+F_{r}+F_{s}\right)\cdot \tau }{J_{\tau }\times ∥\tau ∥}dt,\;J_{\tau }=\sum_{i=1}^{n}m_{i}\times \left(r_{i}^{2}+y_{i}^{2}\right)$$

where *N*_s_ is the number of stators in a particular model, ***r*** is the distance vector from *o* toward the red atom, *τ* is a vector along t-axis. ***F****_s_* is the friction between the rotor and stator along tangent direction of stator. *J_τ_* is the mass moment of inertia of all atoms on rotor about its curved tube axis. “*n*” is the number of atoms on the rotor. When *w_τ_* is non-zero, the rotor will rotate along its curved tube axis.

The rotation of the rotor along the global Y-axis, or the translation of the rotor along the direction of the local axis *oτ*, is essential to be discussed in the present study. The component “***F****_τ_*” produces a linear acceleration of the rotor along its curve tube axis (e.g., the *τ*-axis at the current stator), and the motion direction of the local part of rotor within the stator is aligned with that of ***F****_τ_*. It can also be predicted that the direction is unique on time average in a long enough period (>1000 steps), the average value of ***F****_τ_* is a constant, and particularly, it should be along the positive direction of *τ*-axis. The reason is that the red atom tends to leave away from the IRD atom, and the remaining atoms on stator can also produce repulsion on the atom. Hence, the red atom tends to move along the positive direction of *τ*-axis. The prediction is verified using numerical simulation. The rotor behaves rotating and the rotational frequency can be calculated by

(2) $$\omega \left(t\right)=\omega_{Y}\left(t\right)=N_{S}\times \int_{0}^{t}\frac{R_{Y}\times \left(F_{\tau }+F_{c}\right)\cdot Y}{J_{Y}∥Y∥}dt,\;J_{Y}=\sum_{i=1}^{n}m_{i}\times \left(X_{i}^{2}+Y_{i}^{2}\right)$$

where ***R***_Y_ is the dimension vector from Y-axis toward the red atom. ***F****_c_* is the local intertubular friction which prevents the sliding of the rotor in stator along *τ*-direction. ***Y*** is a vector along the positive direction of Y-axis. *m_i_* is the mass of atom *i* in global coordinates (X*_i_*, Y*_i_*, Z*_i_*). The stable rotational frequency (SRF) of the rotor approaches only when the rotor is at balance state, i.e., ***F****_τ_* = −***F**_c_*.

## 4. Conclusions

The dynamic response of a nanovehicle made from bent CNT is investigated through molecular dynamics simulations. The nanoring can be driven to rotate by the IRD atoms on the stators at room temperature. According to the results with consideration of such core factors as radius of wheel, stator types, IRD atoms, and temperature on the SRF of the rotor, some conclusions are drawn as follows:

(1) For a rotor with small radius, it can be successfully excited to rotate even if the buckling-induced depressions on its surface are heavy. However, the continuous deformation of the depressions needs to overcome the local potential barrier, which results in slower rotation of the rotor.

(2) When the rotor is driven by more short stators, the intertubular friction is smaller because the rotor has smaller curvature near a depression.

(3) When the rotor is confined with the stators after full relaxation, the value of SRF becomes smaller due to weaker collision between the rotor and the stators.

(4) The rotor can be successfully excited to rotate at extremely low temperature, e.g., 8 K. And the SRF of the nanowheel can be regulated by adjusting temperature.

(5) The forward and the backward IRD atoms provide stronger repulsion on the rotor than the internal and external IRD atoms, and result in larger SRF. The more IRD atoms laying uniformly on an edge of each stator, the higher value of SRF of the rotor, but clearly, the SRF of the rotor has a maximum value.

## Figures and Tables

**Table 1:** Geometric parameters of rotors and stators in nanowheels shown in Figure 7.

Ring (i.e., Rotor)				Stator			
Type	Chiral Index	*R*/nm	Num. Atoms	Type	Chiral Index	Length/nm	Num. Atoms
S	(15, 0)	6.373	5640	Short	(24, 0)	0.710	192
M	(15, 0)	12.746	11,280	Long	(24, 0)	6.248	1440
L	(15, 0)	25.493	22,560				
